# Letter to the editor in response to “A 6-year case series of resuscitative thoracotomies performed by a helicopter emergency medical service in a mixed urban and rural area with a comparison of blunt versus penetrating trauma”

**DOI:** 10.1186/s13049-022-01011-7

**Published:** 2022-03-21

**Authors:** E. ter Avest, L. Carenzo

**Affiliations:** 1 Department of Emergency Medicine, University Medical Center Groningen, University of Groningen, PO Box 9713 GZ, Groningen, The Netherlands; 2 Department of Anesthesia and Intensive Care Medicine, IRCCS Humanitas Research Hospital, Rozzano, Milan, Italy

Dear editor

With interest we read the publication of Almond et al. wherein the authors describe a 6-year case series of resuscitative thoracotomies (RT) performed by a helicopter emergency medical service (HEMS) in a mixed urban and rural area in the UK [1]. We’d like to compliment the authors for publication of their (essentially negative) findings, which demonstrates the presence of a very well established governance system.

The authors describe a thoracotomy case series of 44 patients seen by a HEMS service. ROSC was achieved in 11/44 (25%) of the patients attended, but none of the patients survived to hospital discharge. Although the authors mention several factors that could potentially have contributed to their findings, we hypothesize that several other factors may have played a role as well.

First, performing a RT in patients who had signs of life (palpable pulses, respiratory effort) on arrival of the first EMS crew is likely more successful then performing a RT in patients in whom the time of arrest is not confirmed [2]. In this cohort, RT was performed in 17 patients (38%) who had lost signs of life before the arrival of the first EMS crew (on average 9.7 min after the 999 call). HEMS arrived on average 29 [range 15–44] minutes after 999 call. By this time the chances of success of resuscitation efforts would have dropped dramatically, which is reflected by the low ROSC rate in this group (3/17). Therefore, although not absolute, recent guidelines recommend a cut-off of 10–15 min no flow time [3].

Second, in 18 patients the procedure was performed to gain aortic control. Aortic control is not only provided to control bleeding (as mentioned by the authors), but also to facilitate resuscitation of the heart by increasing afterload and thereby coronary perfusion and oxygen delivery to the heart. However, this is only helpful when at the same time oxygenated blood is provided to the coronary arteries by rapid transfusion of packed red blood cells (PRBC’s). As blood products were introduced into the service in 2019, the majority of RT’s (29/44) were performed in a time where mainly crystalloids were available to increase preload. Although crystalloids may help to achieve this, they have no oxygen carrying capacity, and hence their administration under circumstances of traumatic cardiac arrest is unlikely to contribute to ROSC.

Finally, the authors mention that 4/26 RT’s for blunt trauma had a tamponade. Although a tamponade is usually regarded as a treatable cause of arrest, it is important to look at the etiology of the tamponade too. In this study one patient had an aortic arch rupture, one had an LAD-graft rupture, one had a tamponade secondary to abdominal injuries, whilst for the last patient the etiology of the tamponade was not mentioned. These injuries are not treatable in the prehospital setting, and therefore when encountered non-survivable. The relatively high incidence of tamponade by itself should therefore not be regarded as a justification to lower the threshold for RT in blunt trauma patients.

Above all, this very interesting study demonstrates that careful patient selection remains of utmost importance when this procedure is carried out in the prehospital setting. Larger cohort studies are needed to refine indications and contra-indications for this advanced procedure, in particular regarding timelines.

## Data Availability

Not applicable.

## Abbreviations

(H)EMS: (Helicopter) emergency medical service
(P)RBC: Packed red blood cells
ROSC: Return of spontaneous circulation
RT: Resuscitative thoracotomy

## References

[1] Almond P, Morton S, Oeara M (2022). A 6-year case series of resuscitative thoracotomies performed by a helicopter emergency medical service in a mixed urban and rural area with a comparison of blunt versus penetrating trauma. Scand J Trauma Resusc Emerg Med.

[2] Athanasiou T, Krasopoulos G, Nambiar P, Coats T, Petrou M, Magee P (2004). Emergency thoracotomy in the pre-hospital setting: a procedure requiring clarification. Eur J Cardiothorac Surg.

[3] Lott C, Truhlar A, Alfonzo A, Barelli A, Gonzalez-Salvado V, Hinkelbein J, Nolan JP, European Resuscitation Council Guidelines (2021). Cardiac arrest in special circumstances. Resuscitation.

